# Histopathologic Features of Maculopapular Drug Eruption

**DOI:** 10.3390/dermatopathology9020014

**Published:** 2022-03-30

**Authors:** Madison Ernst, Alessio Giubellino

**Affiliations:** 1 Department of Laboratory Medicine and Pathology, University of Minnesota, 420 Delaware St SE, Minneapolis, MN 55455, USA; ernst215@umn.edu; 2 Department of Dermatology, University of Minnesota, 420 Delaware St SE, Minneapolis, MN 55455, USA; 3 Masonic Cancer Center, University of Minnesota, 425 E River Parkway, Minneapolis, MN 55455, USA

**Keywords:** maculopapular eruption, drug eruption, dermatopathology

## Abstract

Background: Cutaneous adverse drug reaction (CADR) is common in both inpatient and outpatient clinical settings and has been associated with a large variety of medications. Drug reactions represent a significant burden to the healthcare system due to increased hospital stay durations and associated costs. Moreover, some of these reactions may be life-threatening. The most common clinical manifestation of a CADR is a maculopapular drug eruption (MDE). Due to its many clinical mimics and associations with a variety of histopathologic patterns, maculopapular drug eruption is difficult to definitively diagnose from both a clinical and histopathological perspective. Summary: We reviewed the clinical and histopathologic features of 327 cases of MDE from several studies in the literature and summarized characteristic histopathologic findings and their frequencies of occurrence. We found that the most common and suggestive histopathologic features of MDE were epidermal spongiosis, lymphocytic infiltrate, and occasional necrotic keratinocytes; interface change at the DEJ; superficial perivascular and interstitial lymphocytic infiltrate with or without eosinophils and neutrophils in the mid-to-deep dermis and mild papillary dermal edema; and dilation of superficial vessels. The presence of multiple histopathologic patterns within the same tissue specimen is also suggestive of MDE. This review and analysis suggest that a biopsy may improve the diagnostic accuracy by both establishing common and uncommon features associated with MDE and reviewing features that help to exclude other causes of maculopapular eruption. Key Message: Histopathologic criteria for the diagnosis of MDE, while not entirely specific, may aid in establishing a differential that includes a drug eruption. Thus, a biopsy can be a helpful diagnostic tool when MDE is suspected by demonstrating findings suggestive of MDE or by ruling out clinical mimics. However, biopsy results cannot be used in isolation as clinical-pathologic correlation is paramount in MDE.

## 1. Introduction

Cutaneous adverse drug reactions (CADR) are a common and costly clinical problem. Reports suggest that CADR affects an average of 2–3% of all hospitalized patients and an even higher number of patients requiring dermatologic consult and biopsy [1]. Although the majority of these cases are not severe or life-threatening, CADRs pose a significant financial burden to the healthcare system, increasing total inpatient medical expenses of affected patients 2.5-fold and total length of hospital stay 1.3-fold compared to inpatients without CADR [2,3]. Since the rate of CADRs correlates with age and polypharmacy, its incidence—and its associated costs—will only continue to grow [2,4,5].

There are many well-characterized clinical subtypes of CADRs that range from mild and otherwise asymptomatic eruptions to more severe diseases involving multiple organ systems, such as Stevens-Johnson syndrome/toxic epidermal necrolysis (SJS/TEN) and drug hypersensitivity syndrome (DRESS). Maculopapular drug eruptions (MDE), also called morbilliform or exanthematous drug eruptions, have long been considered the most common subtype of CADR. Actual reported rates of MDE, however, are dependent on the study population and vary from 30% to 95% of all drug eruptions in the literature [4,5,6].

MDE presents clinically as erythematous macules and/or papules that begin on the trunk and spread symmetrically to the extremities, often coalescing into plaques. This pattern can present alone or within a polymorphous drug reaction, in which MDE presents alongside other CADR patterns, such as urticaria, confluent erythema, or purpura. There are also rare variants of MDE that conform to specific distributions, such as symmetrical drug-related intertriginous and flexural exanthem (SDRIFE). The vast majority of MDEs are mild in clinical severity, with only 0.1% of cases classified as life-threatening [3]. MDE may present as an isolated finding or in association with symptoms such as pruritis and low-grade fever. The eruption typically begins 5–14 days after the causative medication is initiated but may occur weeks to months later. Resolution may occur spontaneously after 1–2 weeks or with cessation of the causative medication [7]. After resolution, desquamation and post-inflammatory hyperpigmentation are common, especially in patients with darker skin tones [7,8].

## 2. Pathophysiology

The pathophysiology of MDE eruptions is incompletely understood and may vary by patient and drug. Most agree that MDE represents idiosyncratic, T-cell-mediated Type IV delayed-hypersensitivity reactions [7,9]. In some cases, antigen-presenting cells present drug haptens—a drug or drug metabolite bound to a larger carrier protein or peptide—to antigen-specific naive CD4+ and CD8+ T-cells [9,10]. This interaction induces a complex immune response involving immune cell proliferation, infiltration of the skin, production of inflammatory mediators (both type I and type II cytokines), and induction of cytotoxicity upon exposure to a drug-protein antigen deposited in the skin [7,11,12,13]. Alternatively, in the p-i (“pharmacologic interaction of drugs with immune receptors”) theory, drugs may trigger an immune response by direct, off-target interaction with immune receptors such as T-cell receptors (TCRs) or major histocompatibility complexes (MHCs) [7,10]. The extreme polymorphic nature of these immune receptors may help explain why both the extent and nature of immunologic response can differ by drug and patient [10]. Regardless of the mechanism, the reaction is not likely to be related to the pharmacologic activity of the causative drug [7].

## 3. Risk Factors

Common risk factors for MDE include polypharmacy, immunosuppression, co-incident infection, systemic autoimmune disease, number of secondary diagnoses, and extremes of age [2]. While viral exanthem is a clinical and histopathologic mimic of MDE, concurrent viral infection increases the risk of maculopapular eruption with the initiation of a drug. For example, nearly 100% of patients with acute Epstein–Barr virus (EBV) will develop a maculopapular exanthem with co-administration of an aminopenicillin antibiotic [7]. Evidence also suggests that concurrent viral infection is associated with more severe drug eruptions and is more likely to have associated systemic symptoms [14]. Immunosuppression may be a risk factor at least in part due to subsequent reactivation of latent infections (i.e., HHV-6, EBV, CMV), which trigger virus-specific CD8+ lymphocytes to secrete IFN-γ and TNFα [14,15].

Several genetic markers have been associated with either higher incidence or increased severity of MDE. These include abacavir and HLA-B5701, carbamazepine and HLA-A3101, and allopurinol and HLA-B5801 [7,16]. These genotypes, however, are uncommon and do not explain the majority of drug eruptions.

MDE can occur with nearly any drug. In fact, most common drugs have cutaneous reaction rates over 1% [3]. High-risk drugs, which are defined as causing MDE in more than 3% of users, include allopurinol, aminopenicillins, cephalosporins, anti-epileptic agents, and antibacterial sulfonamides [7]. Other commonly implicated drugs that do not meet the “high risk” categorization include anxiolytics, nonsteroidal anti-inflammatory drugs, anti-hypertensives, and diuretics [8]. A person’s risk for MDE increases with the number of concurrent medications taken, likely due to drug and metabolic interactions [7]. With the increase in prescription drug use and polypharmacy, the prevalence of MDE is increasing with time [8,17].

## 4. Differential Diagnosis

While drug eruptions are believed to be the most common cause of a maculopapular rash in adults, obtaining a definitive diagnosis of MDE and distinguishing MDE from other CADR that may present with a maculopapular component (e.g., SJS/TEN) can be difficult [7,18]. Guidelines and diagnostic algorithms, such as the Naranjo et al. [19] ARD probability scale and the diagnostic algorithm for severe CADR by Ardern-Jones and Mockenhaupt [20], have been developed to assist in distinguishing drug reactions from non-drug reactions. Few specific tools have been developed to distinguish between CADR subtypes, an important distinction for prognosis and management. Clinically, MDE has a non-specific morphology, distribution, and course. For this reason, MDE may be difficult to distinguish from other cutaneous diseases with maculopapular presentations. A non-exhaustive differential for a maculopapular eruption in adults may include early SJS/TEN, DRESS, acute graft versus host disease (GvHD), viral exanthem, connective tissue disease, allergic contact dermatitis, pityriasis rosea, secondary syphilis, and adult-onset Still disease. In children, maculopapular eruptions are most commonly secondary to infectious processes, such as viral exanthem or bacterial complications (i.e., scarlet fever, mycoplasma pneumonia, and streptococcal or staphylococcal toxin-induced) [7,18]. Less common causes of maculopapular eruption in children include Kawasaki disease, hemophagocytic lymphohistiocytosis, and juvenile idiopathic arthritis. Drug reaction is the inciting cause of rash in 10–20% of pediatric cases [18].

Clinical diagnosis of MDE typically requires the correlation of the cutaneous eruption with drug initiation and observation of symptom resolution after drug cessation. However, this is a difficult task when patients are poor historians, have multiple co-morbidities, or are taking multiple medications. MDE can be diagnosed more definitively by drug re-challenge test, in which recovered patients are administered the suspected causative drug and observed for reoccurrence of the rash. However, this is both impractical and unethical because it exposes the patient to the risk of a more serious reaction upon re-exposure [18].

Clinical clues can be helpful in distinguishing between different CADR. For example, SJS/TEN tends to involve the mucus membranes while MDE does not. DRESS is associated with more severe systemic symptoms, such as myalgias, edema, and lymphadenopathy [7]. For patients with risk factors for multiple conditions, laboratory tests, such as rapid strep, heterophile antibody, rapid plasma reagin (RPR), CBC, and BMP, may be helpful in narrowing the differential. A 2017 study comparing patients with viral exanthem and MDE found that the MDE group had a higher median absolute eosinophil count and serum C-reactive protein [18]. Unfortunately, laboratory tests lack the specificity necessary to provide utility in most cases of maculopapular rash.

The utility of biopsy in maculopapular eruptions is debated. For many years, it was believed that biopsy was only helpful in diagnosing one subtype of CADR: fixed drug eruption [21]. For this reason, the histopathologic features of MDE are often taught as non-specific in dermatology and dermatopathology textbooks [5,12,22,23]. For example, Bolognia’s *Dermatology* [22] emphasizes the “non-specific” histopathology of MDE, describing it only as “superficial perivascular and interstitial lymphocytic infiltrate that may contain eosinophils and interface changes [22,23]”. Rapini’s *Practical Dermatopathology* defines the histopathology of MDE as “perivascular infiltrate of lymphocytes and eosinophils, variable interface dermatitis, and no epidermal change [24]”. Furthermore, texts that do include specific histopathologic features of MDE often lack citations [25].

Given the ambiguous and non-specific histopathologic descriptions of MDE reported in the literature, clinical context remains central to the ultimate diagnosis [11,26]. Although some papers have questioned the utility of performing a biopsy to establish a diagnosis of drug eruption [9,27,28], recent investigations have suggested that certain histopathologic features may be useful to make biopsy an important and potentially lifesaving tool in the context of MDE [6].

One of the most cited early histopathologic characterizations comes from a 1970 report by Fellner and Prutkin that describes the electron and light microscopy findings in four patients with penicillin-related MDE [29]. We were able to find seven additional publications containing information on the histopathologic findings of clinically-confirmed MDE (Table 1). Three of these studies (Naim et al. [8], Gerson et al. [23], and Cho et al. [28]) were aimed at assessing the histopathologic features of only the maculopapular subtype of drug eruption. Two additional studies (Weyers and Metze [6] and Weinborn et al. [29]) reported histopathologic data on various forms of CADR, from which data on maculopapular eruptions were extracted. The last two (Alvarez-Ruiz et al. [30] and Valks et al. [31]) reported rare cases of MDE presenting with a granulomatous histopathologic pattern. In this review, we use these data to collect the histopathologic features of MDE available in the literature and better define useful clues for a correct diagnosis or differential. Common, uncommon, and atypical histopathologic features are compiled in Table 2.

## 5. Histopathologic Features of Maculopapular Drug Eruption

### 5.1. Epidermal Features

One of the most common epidermal features observed among the 327 MDE cases extracted from the literature was mild spongiosis without vesiculation [5,31,32]. Naim et al., who investigated more epidermal features than other studies, found that the spongiotic intercellular edema was typically confined to the lower epidermal layers (78%) and equally likely to be in a focal or continuous distribution. Inflammatory infiltrates were observed in 43–100% of cases in each study and were composed predominantly of lymphocytes and occasional neutrophils. When present, the number and density of the inflammatory cells were low [23]. Intraepidermal eosinophils were rarely seen. Other uncommon epidermal features included parakeratosis, scale crust (a sign of excoriation), compact orthokeratosis, erythrocyte exocytosis, and mitoses.

Mild, regular epidermal hyperplasia, which often occurs secondary to immunologic injury at the DEJ or in conjunction with spongiotic change, was found in an average of 47% of cases [5,31,35]. Severe hyperplasia was never observed. Mild or absent hyperplasia can help distinguish MDE from the moderate to severe epidermal hyperplasia characteristic of psoriasiform dermatoses (i.e., psoriasis vulgaris, chronic atopic dermatitis, and nummular dermatitis) [7]. Psoriasis vulgaris also tends to have confluent parakeratosis, scale crust, and a higher density of neutrophils in the epidermis, features which are uncommon in MDE [36] but may be found in other types of drug eruptions.

The presence of epidermal necrotic keratinocytes, a sign of interface changes, can also be a useful diagnostic feature. While the frequency with which they were reported varied among studies from 21% to 88%, when present, they can help to distinguish MDE from early bullous pemphigoid. The necrotic keratinocytes of MDE are also smaller in number and density than expected in erythema multiforme or toxic epidermal necrolysis [28]. Satellite cell necrosis is not typical in MDE and is more suggestive of GvHD [36]. The absence of Langerhans cell microabcesses and Pautrier microabscesses helps rule out allergic contact dermatitis and mycosis fungoides, respectively [36].

### 5.2. Dermal-Epidermal Junction Features

**Figure 1:**
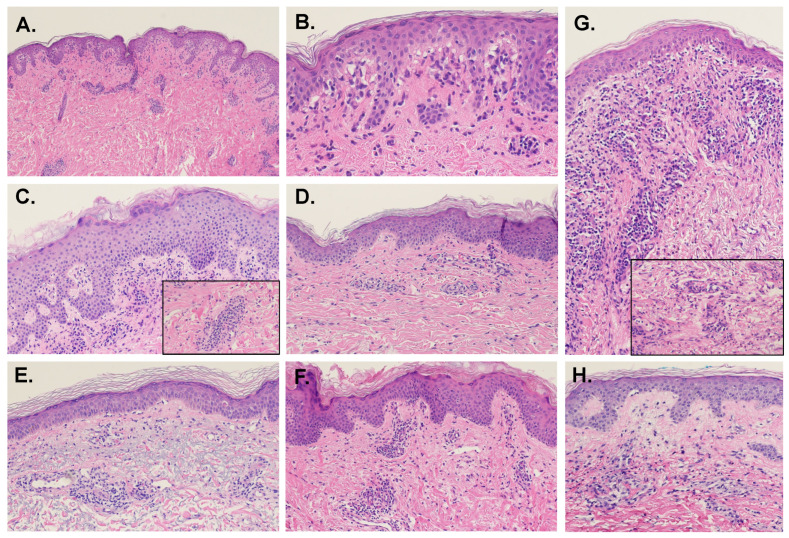
Examples of histopathologic presentations of maculopapular drug eruptions (H&E, 10× magnification; Insets, 20×). (**A**) In this sample, there are interface vacuolar changes above a mild perivascular lymphocytic inflammatory infiltrate. (**B**) Close up of case A: there is evident interface vacuolar alteration with rare dyskeratotic keratinocytes. (**C**) This case presents with more epidermal hyperplasia, mild spongiosis, and perivascular inflammation with eosinophils (inset). (**D**) In this example, there is mild spongiosis, subtle and focal interface changes and mild perivascular inflammation with eosinophils. (**E**) Interface vacuolar changes can be focal, with rare dyskeratotic keratinocytes, and perivascular inflammation. (**F**) This case shows mild epidermal hyperplasia with mild spongiosis, subtle interface changes and mild perivascular inflammation with eosinophils. (**G**) The inflammatory infiltrate with eosinophils (inset) can be more prominent, with interface changes and mild epidermal hyperplasia. (**H**) Mild spongiosis and focal interface and perivascular inflammation with sparse eosinophils characterize this example.

Overall, about 58% of cases were associated with interface vacuolar changes at the DEJ. However, the rates of interface change were not consistent between studies, varying between 27% and 97%. The rates of focal versus continuous distribution of the interface changes also differed by study, with several reports finding the focal pattern more frequent and Naim et al. [8] finding the rates nearly equivalent. Occasional necrotic keratinocytes in the basal layer were found in a third of the cases (Figure 1B). MDE-associated interface change does not typically affect the hair follicles and is not associated with the thickening of the basement membrane.

Vacuolar interface dermatitis is also found in fixed, lichenoid, and erythema multiforme-like drug eruptions. These pathologies are typically associated with more prominent DEJ vacuolization than MDE, as well as a higher density of lymphocytic infiltrate and necrotic keratinocytes at the basal epidermis [7]. Lichenoid drug reaction will also display patchy parakeratosis, compact hyperkeratosis, squamatization of the basal epidermis, dermal colloid bodies, and increased plasma cells [37].

### 5.3. Dermal Features

Inflammatory infiltrate of the superficial dermis was universal. In the majority of cases, the infiltrate was both perivascular (88%) and interstitial (80%). Interestingly, a 2013 paper studying the dermatologic toxicities of certain targeted cancer therapies used the presence of perivascular lymphocytic dermatitis as confirmation that the patient’s maculopapular rashes were caused by a delayed hypersensitivity reaction [38]. The inflammatory infiltrate distribution in MDE was more often patchy than continuous lichenoid. Infiltrate extension into the deep and reticular dermis was uncommon (18%). Cho et al. [28] found that the depth of inflammatory infiltrate in the dermis was significantly greater in antibiotic-associated MDE compared to chemotherapeutic-associated MDE, suggesting that patient comorbidities or the inciting drug may alter the histopathologic findings.

The superficial infiltrate was composed primarily of small, regular lymphocytes. Large lymphocytes were uncommon; however, Naim et al. [8] found them within the perivascular infiltrate in 9 of the 11 cases associated with anticonvulsants and anxiolytics. Frankly atypical lymphocytes were not present in any reported case. Eosinophils and neutrophils were present—usually together—in 57% and 41% of cases, respectively. While eosinophils were more prevalent overall, neutrophils were more likely to be found in the superficial interstitium. Naim et al. [8] also found that neutrophils were significantly more likely to be found in the interstitial infiltrate of MDE associated with anticonvulsants and anxiolytics. The only study to measure macrophage presence found them to be relatively common (65%) in the superficial dermis. However, granuloma formation was not observed. The number of mast cells was not altered. In contrast with the lymphocytic predominance of the superficial dermal infiltrates, deep dermal infiltrates were predominantly eosinophilic and neutrophilic with only half of the cases showing small, regular lymphocytes. Examples of MDE histopathology are shown in Figure 1.

### 5.4. Connective Tissue and Vasculature

Other than the dilation of the small vessels of the superficial vascular plexus, vascular changes such as fibrinoid degeneration of vessel walls, thrombi, and leukocytoclasia were absent in MDE. Erythrocyte extravasation was uncommon (28%). Dilation of superficial dermal lymph vessels was nearly universal. Edema of the superficial papillary dermis was a common finding (69%). Extension into the deep and reticular dermis was less common (43%) and typically remained mild and perivascular.

Interstitial mucin deposits were absent, which can differentiate MDE from other interface dermatoses, such as SLE, dermatomyositis, scleromyxoedema, and papular mucinosis [36]. MDE also lacked epidermal atrophy, the presence of which would favor connective tissue disease [11].

### 5.5. Presence of Eosinophils

The presence of eosinophilic infiltrate has long been used as a histopathologic sign for differentiating drug eruption from microscopically similar pathologies, such as viral exanthem, GvHD, and lupus erythematosus. Only 62% of the MDE cases we reviewed contained eosinophils. When present, they were found within the primarily lymphocytic, superficial dermal infiltrate. They were not typically found in the epidermis or at the DEJ. However, neither the presence nor patterning of eosinophils is sensitive or specific for MDE. While eosinophils are less common in other pathologies, they can still be found in 3–5% of GvHD and viral exanthems [35,39]. An over-reliance on eosinophilic presence has been the subject of many papers on the histopathologic diagnosis of MDE due to the significant morbidity of patients who receive delayed care after misdiagnosis, especially in cases of missed GvHD [35]. However, one paper has suggested that the quantification of eosinophils, instead of the mere presence, may be sufficient to distinguish acute GvHD and MDE: their study found that a very high number of eosinophils—an average of 16 per 10 high-power fields (HPFs)—effectively rules out acute GvHD [40].

## 6. Variants of Maculopapular Drug Eruption

### 6.1. MDE with Urticarial Aspect

One study estimates that 15% of all MDE have an additional urticarial aspect, distinct from true, drug-induced urticaria [8]. Clinically, MDE-associated urticaria is less pruritic and lasts longer than true urticaria. MDE-associated urticaria may also have scale. Histopathologically, lesions of MDE with and without urticarial aspect do not show any consistent differences. The predominant pattern in both is interface dermatitis with epidermal spongiosis. The severity of edema is similar. These findings are distinctly different from the classic histopathology of urticarial drug reaction, which is characterized by an isolated finding of dermal edema with separation of collagen fibers of the reticular dermis [41].

### 6.2. MDE with Granulomatous Change

While granulomatous inflammatory patterns are rarely caused by drug reactions, there have been several reports of MDE with granulomatous histopathology occurring in patients 1 day to 3 weeks after receiving granulocyte colony-stimulation factor (G-CSF) or GM-CSF. Histopathologically, these cases have many of the same findings as non-granulomatous drug eruption: mild vacuolar interface dermatitis and spongiosis, perivascular lymphocytic infiltrate in the papillary dermis, mild dermal edema, and dilated blood vessels. However, instead of the predominantly lymphocytic interstitial infiltrate typical of MDE, the samples show a variable mixture of granulocytes, enlarged macrophages, and regular lymphocytes throughout the interstitium of the papillary and occasional reticular dermis [42]. These samples also show an increased number of histiocytes [30,31].

### 6.3. MDE Due to Targeted and Other Oncologic Therapies

Despite having more “specific” targets than traditional chemotherapies, targeted oncologic agents are frequently associated with off-target CADR [43]. The subtype, presentation, and histopathologic features of the associated CADR are dependent on the specific agent used. MDE is associated most commonly with certain agents within the following classes: KIT and BCR-ABL inhibitors, multikinase inhibitors, antimetabolites, mitotic/spindle inhibitors, and mTOR inhibitors. Treatment for targeted therapy-associated MDE is with topical or oral steroids, and agent discontinuation is rarely warranted [43]. Since targeted therapies have also been associated with rare yet severe diseases requiring specific management, such as SJS, being able to distinguish MDE histopathologically from these diagnoses may be of critical importance [44].

As an example, KIT and BCR-ABL inhibitors, such as imatinib, and nilotinib, and dasatinib, are associated with MDE, edema, and pigmentary changes that begin an average of 9 weeks after treatment initiation [43]. Histopathology of imatinib-associated MDE is similar to that of general MDE discussed above with epidermal parakeratosis, spongiosis and necrosis of keratinocytes, DEJ irregularity, papillary dermal edema, and lymphocytic and histiocytic infiltrate [44,45].

Multikinase inhibitors, including sorafenib, sunitinib, pazopanib, MK-2206, and vemurafenib are most strongly associated with inflammatory CADR. The most common is MDE, which begins on the face and spreads centripetally [43]. Histopathology is characterized by mild perifollicular lymphocytic infiltrate with occasional eosinophils [38,46]. A similar histopathologic presentation has been reported with antimetabolite-associated MDE [47]. MDE of the mTOR inhibitors everolimus, temsirolimus, and ridaforolimus also begins on the face within the first weeks of treatment [48]. On biopsy, histopathology demonstrates non-specific dermal and epidermal neutrophilic infiltrate [49].

MDE associated with mitotic/spindle inhibitors, including vinca alkaloids, taxanes, podophyllin, and their derivatives, exhibit the most distinctive histopathology due to the mechanism of the drug on mitotic arrest. On biopsy, these MDEs typically show epidermal dysmaturation with large, atypical keratinocytes, atypical intraepidermal mitoses with characteristic “starburst” or ring-like cells, and epidermal apoptosis and necrosis [50]. Careful histopathologic interpretation may be necessary for certain situations as individual lesions can histologically mimic carcinoma in situ [50].

## 7. Conclusions

MDE is a common condition with many clinical mimics and frequently represents a challenging diagnosis. We have reviewed the histopathologic findings reported in 327 cases of MDE from eight different papers in the literature and presented an overview of the histopathologic features that, while perhaps not entirely specific, may be suggestive of drug eruption. Such features include epidermal spongiosis, mild lymphocytic infiltrate, and occasional necrotic keratinocytes; interface change at the DEJ; superficial perivascular and interstitial lymphocytic with or without eosinophils and neutrophils in the mid-to-deep dermis and mild papillary dermal edema; and dilation of superficial dermal lymph and blood vessels. Moreover, it is important to emphasize that a drug eruption should always be considered in the differential when multiple histopathologic patterns—none of which conform to another precise diagnosis—are present within the same tissue section. While histopathology may not always be entirely specific and must be interpreted with careful consideration of clinical correlation, we conclude that biopsy may provide critical utility in the diagnosis of maculopapular eruptions by helping to either include or exclude specific etiologies.

## Figures and Tables

**Table 1:** Histopathologic features of 327 cases of maculopapular drug eruption from the literature.

Feature	Weinborn et al. [29] *n* = 33 *n* (%)	Naim et al. [8] *n* = 60 *n* (%)	Cho et al. [28] *n* = 40 *n* (%)	Gerson et al. [23] *n* = 104 *n* (%)	Bellini et al. [32] *n* = 36 *n* (%)	Ortonne et al. [33] *n*= 20 *n* (%)	Wang et al. [34] *n* = 10 *n* (%)	Signh et al. [18] *n* = 24 *n* (%)	Overall *n* = 327 %
**Epidermis**									
Hyperplasia	-	43 (72)	18 (45)	-	9 (25)	-	-	5 (21)	47
Basket-weave orthokeratosis	-	-	19 (48)	-	23 (64)	-	-	-	55
Compact orthokeratosis	-	8 (13)	-	-	-	-	-	2 (8.3)	12
Scale crust	-	5 (8)	-	-	-	-	-	-	8
Parakeratosis (any)	6 (18)	10 (17)	5 (13)	-	3 (8.3)	8 (40)	-	2 (8.3)	16
Focal	-	8 (13)	-	-	3 (8.3)	-	-	-	11
Compact	-	2 (3)	-	-	-	-	-	-	3
Spongiosis present (any)	23 (70)	58 (97)	21 (53)	-	22 (61)	-	-	12 (50)	70
Lower only	-	47 (78)	-	-	-	-	-	-	78
All Layers	-	11 (18)	-	-	-	-	-	-	18
Focal	-	27 (45)	-	-	-	-	-	12 (50)	46
Continuous	-	31 (52)	-	-	-	-	-		52
Inflammatory infiltrate (any)	14 (42)	60 (100)	27 (68)	-	17 (47)	7 (35)	9 (90)	14 (58)	66
Lymphocytic	-	49 (82)	25 (63)	-	17 (47)	7 (35)	-	14 (58)	62
Neutrophilic	-	19 (32)	2 (5)	-	7 (19)	-	-	1 (4.2)	18
Eosinophilic	0	2 (3)	3 (7.5)	-	-	-	-	2 (8.3)	4
Erythrocytes	-	5 (8)	-	-	-	-	-	2 (8.3)	16
Necrotic keratinocytes	8 (24)	13 (22)	35 (88)	-	-	-	-	5 (21)	39
Atrophy	8 (24)	-	-	-	-	-	-	-	24
Mitoses	-	20 (33)	-	-	-	-	-	2 (8.3)	26
DEJ									
Basal vacuolization/ interface change (any)	9 (27)	58 (97)	38 (95)	54 (52)	11 (31)	7 (35)	7 (70)	7 (29)	58
Focal	-	26 (43)	29 (73)	-	-	7 (35)	-	6 (25)	47
Continuous	-	32 (53)	9 (23)	-	-	-	-	1 (4.2)	34
**Dermis**									
Edema (superficial)	20 (61)	51 (85)	26 (65)	-	26 (73)	7 (35)	-	-	69
Edema (deep)	-	28 (47)	1 (2.5)	-	11 (29)	-	-	-	43
Infiltrate (any)	33 (100)	60 (100)	40 (100)	102 (98)	33 (92)	20 (100)	-	15 (62)	96
Superficial (any)	33 (100)	60 (100)	40 (100)	97 (95)	-	20 (100)	-	-	97
Deep (any)	9 (27)	29 (48)	2 (5)	5 (5)	-	1 (5)	-	-	18
Perivascular (any)	-	60 (100)	-	102 (98)	33 (92)	8 (40)	-	12 (50)	88
Superficial Perivascular (any)	-	43 (72)	-	-	-	-	-	-	72
Deep Perivascular (any)	-	17 (28)	-	-	-	-	-	-	28
Interstitial (all)	-	56 (93)	-	82 (80)	-	-	-	13 (54)	80
Superficial	-	56 (93)	-	-	-	-	-	-	93
Deep	-	29 (48)	-	-	-	-	-	-	48
Lymphocytic (all)	-	60 (100)	40 (100)	95 (91)	-	20 (100)	-	14 (58)	92
Perivascular	-	60 (100)	-	-	-	-	-	12 (50)	86
Interstitial	-	53 (88)	-	-	-	-	-	2 (8.3)	65
Superficial	-	60 (100)	-	-	-	-	-	14 (58)	88
Deep	-	29 (48)	-	-	-	-	-	-	48
Eosinophilic (all)	20 (61)	46 (77)	27 (68)	52 (50)	13 (36)	9 (45)	6 (60)	15 (62)	57
Perivascular	-	36 (60)	-	-	-	-	-	7 (29)	51
Interstitial	-	33 (55)	-	-	-	-	-	13 (54)	55
Superficial	20 (61)	36 (60)	-	-	-	-	-	2 (8.3)	60
Deep	4 (12)	46 (77)	-	-	-	-	-	-	54
Neutrophilic (all)	-	38 (63)	11 (28)	37 (36)	-	6 (30)	-	2 (8.3)	41
Perivascular	-	30 (50)	-	-	-	-	-	1 (4.2)	50
Interstitial	-	46 (77)	-	-	-	-	-	1 (4.2)	77
Superficial	-	46 (77)	-	-	-	-	-	1 (4.2)	77
Deep	-	38 (63)	-	-	-	-	-	-	63
Macrophages	-	39 (65)	-	-	-	-	-	-	65
Erythrocytes	-	17 (28)	-	-	-	-	-	-	28

**Table 2:** Summary of histopathologic findings in MDE by commonality.

	Common (>55%)	Less Common (26–55%)	Atypical Findings (<25%)
Epidermis	Focal or continuous spongiosis of lower epidermis without vesiculation	Mild, regular hyperplasia	Discrete mounds of parakeratosis
	Mild lymphocytic infiltrate	Necrotic keratinocytes	Compact orthokeratosis
	Regular basket-weave orthokeratosis	Mitoses	Increased eosinophils, neutrophils, melanophages, or erythrocytes
			Pronounced epidermal damage
			Scale crust
			Satellite cell necrosis
			Langerhans cell microabscesses
DEJ	Basal vacuolization/interface change (focal or continuous)	Necrotic keratinocytes	
Dermis	Superficial perivascular and interstitial lymphocytic and eosinophilic infiltrate +/− neutrophils	Erythrocyte extravasation	Colloid bodies
	Macrophages without granuloma formation	Deep, interstitial and perivascular infiltrate	Atypical lymphocytes
			Increased mast cells
Connective tissue and vessels	Mild edema of papillary dermis		Vasculitis/leukocytoclasia
	Dilation of superficial dermal lymph and blood vessels		Fibrosis
			Interstitial mucin deposits
